# Prevalence and reasons to seek traditional healing methods among residents of two localities in North Kordofan State, Sudan 2022: A cross‐sectional study

**DOI:** 10.1002/hsr2.1487

**Published:** 2023-08-22

**Authors:** Ghassan E. Mustafa Ahmed, Exeer Yahia M. Ahmed, Ayat Eltahir Ahmed, Lina Hemmeda, Anmar B. Birier, Tibyan Abdelgadir, Hadiea Mosaab AhmedElbashir Hassan, Esraa S. A. Alfadul, Musab Bakr, Ethar Sadig

**Affiliations:** ^1^ Faculty of Medicine University of Khartoum Khartoum Sudan

**Keywords:** health seeking, North Kordofan, Sudan, traditional medicine

## Abstract

**Background and Aims:**

Traditional medicines are one of Africa's primary response mechanisms to medical emergencies, while, in other communities, all healthcare decisions are based on indigenous traditions and beliefs. For millions of individuals who reside in rural areas throughout low‐ and middle‐income nations, those healers serve as their only primary healthcare practitioners. This study intends to examine the availability, preferences, and practices of traditional medicine seeking among the Sudanese of North Kordofan state.

**Method:**

A descriptive cross‐sectional study was carried out in two conveniently chosen localities in North Kordofan state, namely Barrah, and Shaikan. A structured questionnaire with three sections—sociodemographic, attitudes, and forms of traditional medical practices—was used for face‐to‐face interviews with the residents. The frequency (*n*) and percentage (%) of categorical data are presented. The Chi‐square and Fischer exact tests were performed to determine characteristics related to traditional medicine practice and the preferred type of medicine among participants; a *p* value of.05 was considered significant.

**Results:**

A total of 302 residents took part in the study, with the 15–30 age group constituting the majority. The majority of participants (89.1%) used traditional medicine, and the majority of them (92.4%) learned about it from their families. The major type of traditional medicine (60.3%) used was a medical herbs–herb practitioner. Cultural influences (57%) and the effectiveness of traditional medicines (48.3%) were the most common reasons for seeking those medicines.

**Conclusion:**

Most participants seek traditional medicine, with traditional herbs and holy recitation commonly used. Affordability, therapeutic effectiveness, and cultural and religious influence were reasons for preferring traditional medicine.

## 1 INTRODUCTION

Traditional medicine, as defined by The World Health Organization (WHO), is the indigenous knowledge, skill, and practices used for maintaining and improving physical and mental health.^1^ In contrast to modern medicine, traditional healing methods are culturally accepted and holistic, making them a good choice for the promotion of health care in a community.^2^ Traditional healer, on the other hand, uses natural remedies as well as social, cultural, and religious practices to provide health care to the community where he or she lives. ^3^

The traditional medical system incorporates several healing methods: medicinal plants, animal products, minerals, and physiotherapy.^4^ According to the WHO, herbal remedies are the most common type of traditional medicine, with 70%–80% of people in the African region using them as their primary health care. This can be attributed to the fact that most herbs used for traditional medicines are grown near homesteads and are, by and large, free of charge.^5^ In Sudan, a wide variety of traditional healing practices are encompassed. According to El Safi et al., traditional medicine is divided into two categories: general practitioners and specialists. Religious healers and “magic‐mongers” are general practitioners, while Herbalists, Zar practitioners, Baseers (bonesetters), Dayas (midwives), and Shallaqs (eye surgeons) are specialists. The religious healers use some religious procedures such as incantations (al‐ruqia) and the eraser (al‐mihaya). On the other hand, herbalists rely on Sudan's rich medical recipes, which comprise a wide range of recipes made from medicinal plants for therapeutic, nutritional, health promoting, preventative, and cosmetic purposes. Some of the commonly used herbals in Sudan are haraz (*Acacia albida*) for diarrhea; harjal (*Solenostmma argel*) for headache, fever, and common cold; hernab (*Carissa edulis*) as antifungal; hilba (funegreek) for epigastric pain, joint affliction, and abdominal disorders; garad (*Acacia nilotica*) for fever and sore throat and simbil (*Andropogon nardus*) for fever and as an anti‐inflammatory and demulcent.^6^

As to the WHO, 65%–80% of the world's healthcare practice includes the use of traditional medicine in some way.^7^ In Africa, traditional medicine is part of the first set of response mechanisms for medical emergencies, whereas, in other communities, all healthcare decisions are based on indigenous practices and beliefs. Traditional healers are said to be the only primary healthcare providers for millions of people living in rural areas in various low‐ and middle‐income countries. For example, the traditional health professionals‐to‐population ratio is 1:500, while the physician‐to‐population ratio is 1:40,000.^8^ Several factors have been identified as responsible for the widespread use of traditional medicine, most commonly the alignment of traditional medicine with the patient's sociocultural, religious, and spiritual values. Furthermore, traditional healers are trustworthy as their patients share with them their secrets. In addition, the low cost, the flexibility of payment, and the accessibility drive the use of traditional medicine.^9^

In Sudan, the low level of health education and socioeconomic status among patients, meager health infrastructure, and lack of trained healthcare workers, particularly in rural areas, have contributed to the extensive use of traditional medicine.^10^ A study has shown that traditional healing is the most common approach for treating patients with mental illnesses in Sudan, owing to a lack of financial resources, the inaccessibility of medical facilities, and a lack of public knowledge.^11^ Another study in Sudan has shown that of the hundred interviewed families 70% were using traditional treatments, causing delays in presentation to hospital in 24% of children ***.*** ^12^ Recent studies have discussed the role of traditional medicine in the attainment of Universal Health Coverage (UHC).^13^, ^14^

Despite the benefits of traditional medicine and its wide use, traditional healing is not fully institutionalized since there is no accountable government organization that leads and regulates the performance of traditional healing services.^5^ Another significant issue is the lack of a reference standard for calculating the right dosage of conventional medication for patients. Subsequently, this has led to the modeling of inaccurate and insufficient traditional medicinal drugs.^15^ Up to our knowledge, no studies were conducted in Sudan regarding the prevalence and reasons for seeking traditional healing methods in North Kordofan. Consequently, there is insufficient data to establish policies that would regulate traditional medicine practices and improve overall healthcare services. This is especially critical in a country where healthcare professionals are in short supply and their distribution is uneven. Therefore, in this study, we aim to analyze the traditional medicine‐seeking prevalence, choices, and practices among the residents in the state of North Kordofan.

## 2 METHODS AND MATERIALS

**Figure 1:**
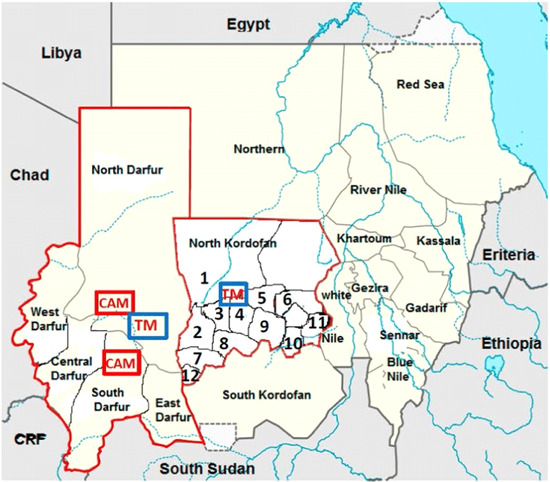
Map of Sudan and the region of North Kordofan State. https://images.app.goo.gl/Gb5khCNzW7DsJfsw9

This is a descriptive cross‐sectional study, carried out from March to July 2022, and conducted in North Kordofan state as part of the medical mission project conducted by Khotwa Charity Foundation. North Kordofan State lies between latitudes 11°15′–16°45′N and longitudes 27°5′–32°15′E. North Kordofan state has an area of 185,302 km² and a population of 2,760,441.^1^ The state is bounded by the Northern state from the north, Khartoum State and White Nile state from the east, and South Kordofan state from the south. The state is divided into administrative units called localities as in Figure 1. Two localities were conveniently selected from the state, they are namely Barrah and Shaikan, which are numbered 6 and 9 in Figure 1, respectively. These two localities were selected based on the fact that medical practitioners and health services are scarce when compared to nearby localities, along with the scarcity of medications. The targeted residents of Barrah and shaikan were 15 years and above. The sample size was collected using the equation (*n* = *N*/1 + N*(*e*)2) = 399. The form of data collection was directed by using author‐designed structured questionnaires that were used to ask the participants face‐to‐face in interviews. The questionnaire contained three sections: socio‐demographic information, attitude and practices, and types of traditional methods were preferred. We chose equal numbers of males and females randomly; they all were 333 participants (83% of the sample size). The questionnaire was translated into the Arabic language to facilitate the questions and to be realizable for the participants. Data collectors were trained by the study authors before doing the interviews and they entered the data in English by using an offline smartphone collection application (Kobo Collect). Data were initially entered and cleaned using Microsoft Excel, then analyzed using Statistical Package for Social Sciences (SPSS) version 25.0, and categorical data presented in the form of frequencies (*n*) and percentages (%). For analytical statistics, Chi‐square and Fischer exact test were used to identify factors associated with the practice of traditional medicine and preferable type of medicine among participants, a *p* value of <0.05 was considered significant.

## 3 RESULTS

This study included a total of 302 participants from two localities in North Kordofan state: Bara (36.3%) and Shikan (63.3%). The most common age group was 15–30 years (41%), males to females’ ratio was nearly equal. All participants were Muslims, and most of them were married (67.5%) and almost half of them (48%) received primary education. The most common occupation among participants was (farmer) (37.4%) with an average family monthly income of 50,000–100,000 SDG (Table 1).

**Table 1:** Sociodemographic data of the participants.

Characteristics	Classification	Total *N*	%
Locality	Bara	109	36.3
	Shikan	190	63.3
Age groups (years)	15–30	124	41.1
	31–45	91	30.1
	46–60	46	15.2
	61–86	41	13.6
Gender	Male	153	50.7
	Female	149	49.3
Religion	Muslim	302	100.0
Marital status	Single	93	30.8
	Married	204	67.5
	Divorced	3	1.0
	Widowed	2	0.7
Educational level	Illiterate	78	25.8
	Primary school	145	48.0
	Secondary school	55	18.2
	University	24	7.9
Occupation	Farmer	113	37.4
	Government worker	1	0.3
	Housewife–Husband	78	25.8
	Daily worker	52	17.2
	Military	6	2.0
	Employee	1	0.3
	Student	26	8.6
	Midwife	3	1.0
	Teacher	10	3.3
	No work	11	3.6
Monthly family income	Less than 50,000 SDG	115	38.1
	50,000–100,000 SDG	163	54.0
	101,000–150,000 SDG	11	3.6
	More than 150,000 SDG	13	4.3

The family was the main source of information about traditional medicine use (92.4%) followed by friends (37.5%), most of the participants in this study practiced traditional medicine (89.1%) in an irregular pattern\when unwell (94%), and after informing a physician in almost half of cases. The outcome of using traditional medicine is symptomatic relief in 89.4%; the most preferable type of medicine was modern medicine in almost two‐thirds of participants (69.9%). Further details are shown in Table 2.

**Table 2:** Practice of traditional medicine (total *n* 302).

Sources of information about traditional medicine use	*N*	% of cases
Family	278	92.4
Friends	113	37.5
Healthcare practitioners	10	3.3
Newspaper	5	1.7
Television	7	2.3
Radio	30	10.0
Internet	2	0.7

Did you ever practice traditional medicine	*N*	%
Yes	269	89.1
No	33	10.9

How often do you use traditional medicine	*N*	%
Regularly	18	6.0
Irregular/when unwell	284	94.0
If regularly, how frequently	*N*	%
Daily	9	50
Weekly	3	16.7
Once a month	2	11.2
Once every 3 months	2	11.2
Once every 6 months	1	5.6
Once a year	1	5.6

Do you inform a physician before taking traditional medicines	*N*	%
Yes	175	57.9
No	127	42.1

Outcome of using traditional medicine for management of health problems	*N*	%
Symptomatic relief	270	89.4
Permanent cure	16	5.3
No change	12	4.0
Harm	4	1.3

By whom traditional medicine was practiced	*N*	% of cases
Traditional medicine healer	30	9.9
Elderly	142	47.0
Myself	221	73.2

Which type of medicine do you prefer	*N*	%
Traditional medicine	16	5.3
Modern medicine	211	69.9
Both	74	24.5

Regarding the practice of traditional medicine, the most common health problems for which is being practiced are cold, influenza and cough (84.8%), diarrhea, stomach, and intestinal infections (63.2%); different types of traditional medicine are being used but medical herbs–herb practitioner (60.3%) was the most popular in this study. Reasons for seeking traditional medicine methods also varied; cultural influences (57%) and the effectiveness of traditional medicines (48.3%) were the most common reasons. More details are shown in Tables 3, 4, 5).

**Table 3:** Health problems to which participants practice traditional medicine.

Health problem	*N*	% of cases
Cold–influenza–cough	256	84.8
Headache	104	34.4
Fever	89	29.5
Diarrhea–stomach and intestinal infections–poisoning	191	63.2
Injuries‐burns	68	22.5
Animals’ bites—venom	41	13.6
Eye diseases	49	16.2
Paralysis	2	0.7
Skin diseases	70	23.2
Spiritual possession—demon	2	0.7
Diabetes	31	10.3
infertility—gynecological problems	4	1.3
STDs	47	15.6
Others	2	0.7

Abbreviation: STD, sexually transmitted disease.

**Table 4:** Common types of traditional medicine used.

Type	*N*	% of cases
Medical herbs–herb practitioner	182	60.3
Faith healer–holy water–oils	156	51.7
Sheikh for reciting Quran	72	23.8
Cautery	67	22.2
Cupping	58	19.2
Sheikh to expel jinn spiritual	3	1.0
Bonesetter	82	27.2
Camel milk	98	32.5
Zaar	2	0.7
Headband	16	5.3
Midwife	140	46.4
Massage therapy	12	4.0
Exercise for relaxation–meditation–yoga	2	0.7
Sudanese recipes (Garad, karkade, Helba, Laurel, Aradib, Gadim, Tabaldi, Hargel, Sidra, Wadek)	104	34.4
Others (Cumin, Gum, Limon, Clove, Orange, Coal, Custard, Salt, Sugar, Millet)	31	10.3

**Table 5:** Reasons for seeking traditional medicine.

Reason	*N*	% of cases
Religious affiliations	19	6.3
Cultural influence	172	57.0
Effectiveness of traditional medicines	146	48.3
Preference of natural materials	31	10.3
Affordability—expensive fees of physician	85	28.1
Distant health facilities	86	28.5
Non response to medical treatment	16	5.3
Dissatisfaction with physician's diagnosis	2	0.7
Remote appointment for specialist or consultant	6	2.0
Long waiting for consultation of physicians	5	1.7
Expensive drugs	49	16.2
There are fewer side effects of traditional medicine	4	1.3
Traditional medicines build up the body's own defenses and promotes self‐healing	3	1.0
Traditional medicines are comfortable because they don't need different test reports which doctors ask for diagnosis	11	3.6
Traditional healers use methods which help psychologically	1	0.3
Traditional medicines are totally harmless	4	1.3
Addiction to traditional medicines products	8	2.6
Traditional healers provide better communication	4	1.3
Traditional healers spare more time for patients	45	14.9
No specific reason	4	1.3

### 3.1 Factors related to practice and preference of traditional Medicine

Characteristics of participants found to be significantly related to the practice of traditional medicine are locality, age, gender, educational level, and income, while those related to the preferable type of medicine are locality, age, gender, and monthly income. (Table 6).

**Table 6:** Practice and preference of traditional medicine by participants according to their characteristics (*n* 302).

Sociodemographic characteristics	Practice			Preference			
	Yes	No	*p* Value	TM	MM	Both	*p* Value
Locality							
Bara	90	19	0.02	6	57	45	0.000
Shikan	176	14		10	151	29	
Age group (years)							
15–30	117	7	0.04	7	96	21	0.003
31–45	75	16		8	54	29	
46–60	40	6		0	32	14	
61–86	37	4		1	29	10	
Gender							
Male	153	0	0.000	9	141	3	0.000
Female	116	33		7	70	71	
Educational level							
Illiterate	62	16	0.01	5	45	27	0.07
Primary school	133	12		6	107	32	
Secondary school	52	3		5	42	8	
University	22	2		0	17	7	
Monthly income							
Less than 50,000 SDG	110	5	0.006	7	98	10	0.000
50,000–100,000 SDG	136	27		9	91	62	
101,000–150,000 SDG	10	1		0	9	2	
More than 150,000 SDG	13	0		0	13	0	

Abbreviations: MM, modern medicine; TM, traditional medicine.

## 4 DISCUSSION

This study was conducted in two localities in North Kordofan state, Sudan. The main issues tackled by this study were the prevalence of traditional medicine use in North Kordofan, along with the traditional methods adopted and the common reasons for seeking traditional medicine.

A sample of 302 residents—aged between 15 and 86 years—participated in this study, with the prevalence of traditional medicine use estimated as 89.1%, which is higher than that estimated by a study conducted in Riyadh, Saudia Arabia in 2010 (42%)^16^ and also higher than a study conducted in south‐west Ethiopia, 2021 (81.5%).^17^

Most of the participants reported they prefer modern medicine to traditional methods (69.9%), which can be justified by the finding that symptomatic relief—not cure—is the main outcome of using traditional medicine (89.4%). However, the high prevalence of traditional medicine users and the self‐practice of traditional medicine without an authentic prescription (73.2%) is owed to the lack of efficient health care and health centers in the region. These findings were similar to those discovered in a study conducted in south‐west Ethiopia, in 2021, which found the majority prefers modern medicine (75%), but seek traditional medicine as a result of affordability issues, as well as religious issues,^17^ which is apparent in various rituals. A meta‐analysis was conducted on 200 publications on the uses of African palms and found information about ritual uses in 26 publications. It was reported that in some rituals, palms play a sacred role as holy objects, for example, the seeds accompanying oracles and palm leaves are used in offerings. On other occasions, palms are added as a support to other medicinal ingredients, for example, palm oil is used as a medium to blend and make coherent the healing mixture.^18^

Respiratory health problems, such as influence and common cold, followed by gastrointestinal conditions, such as diarrhea and infections, were the major health problems for which traditional medicine is adopted. These findings are in agreement with the Ethiopian study, with cough, headache, and abdominal conditions being the most common health problems opposed by traditional medicine,^17^ as well as a study conducted in northern Rwanda,^19^ but in contrast to the Saudi study in Riyadh which reported abdominal pain and back pain as the main condition for which traditional methods are adopted.^16^ In a similar study, the analysis revealed that the use of traditional medicine ranged from chronic conditions, complex spiritual or psychosocial problems, mental illness, chronic conditions, acute conditions, generalized pain, HIV, and other sexually transmitted diseases.^20^

Cultural influences, effectiveness, and affordability were the most reported reasons behind seeking traditional medicine, which gives an insight into the socioeconomic condition in the study area. These are similar reasons to those reported by previous studies in Ethiopia, Rwanda, and Saudi Arabia, all agreeing that lack of affordable health care, as well as the perceived benefits from traditional medicine use, were the major reasons for seeking traditional medical methods.^16^, ^17^, ^19^ This is also compatible and supported by the findings of a previous study among pregnant African women, which revealed that frequent traditional medicine users were pregnant women with no formal education, low income, and living far from public health facilities.^21^

Regarding the most common traditional methods adopted, medical herbs were the most reported and used by study participants followed by recitation of holy verses, which could be owed to the previously mentioned cultural and religious influence. The Saudi study conducted in Riyadh, and the Ethiopian study, reported similar adopted methods.^16^, ^17^ Other methods adopted were cautery, drinking camel milk, and traditional Sudanese herbs.

These results indicate that traditional healing methods are prevalent and still play a major role in the individual's health‐seeking behavior, especially when it is linked with a religious background, as reported in a previous study in Bangladesh, that people of low socioeconomic status first approach the traditional healers with their medical problems who resort to religious rituals, and usually used verses of holy books in healing.^22^

In addition, the harmful potential of traditional healers lies on the fact that they do not receive authentic academic education in medicine, which is supported by responses of a previous study where traditional healing practitioners were asked about how they diagnose illnesses and majority responded they diagnose illnesses through various approaches including consultations with spirits, observing patterns of occurrences and events, use of bones from animals/birds and other objects to diagnose illnesses, and performing diagnostic rituals.^23^

The strength of this study is that it is the first study to tackle traditional medicine in the region of North Kordofan, including participants of both genders, various age groups, and cultural and religious beliefs, which we believe that the findings reported would be of benefit towards better healthcare distribution and would be the sparkle to focus the light on the issue of traditional medicine in Sudan as a whole.

The limitation of this study is that it only included two localities out of three in North Kordofan State, as well as it did not question the actual effectiveness of the traditional methods adopted, as well as it did not include opinions from medical practitioners in the area.

## 5 CONCLUSION

The majority of the participants seek traditional medicine, with traditional herbs and holy recitation being the commonly used methods. Affordability, therapeutic effectiveness, and cultural and religious influence were reasons for preferring traditional medicine.

## AUTHOR CONTRIBUTIONS

**Ghassan E. Mustafa Ahmed**: Conceptualization; data curation; methodology; project administration; supervision; writing—original draft; writing—review and editing. **Exeer Yahia M. Ahmed**: Conceptualization; data curation; formal analysis; methodology; project administration. **Ayat Eltahir Ahmed**: Conceptualization; data curation; methodology; writing—original draft. **Lina Hemmeda**: Conceptualization; methodology; writing—original draft. **Anmar B. Birier**: Conceptualization; data curation; formal analysis; writing—original draft. **Tibyan Abdelgadir**: Conceptualization; data curation; methodology; writing—original draft. **Hadiea Mosaab AhmedElbashir Hassan**: Conceptualization; writing—original draft. **Esraa S. A. Alfadul**: Writing—original draft; writing—review and editing. **Musab Bakr**: Writing—original draft; writing—review and editing. **Ethar Sadig**: Conceptualization; writing—review and editing.

## CONFLICT OF INTEREST STATEMENT

The authors declare no conflict of interest.

## ETHICS STATEMENT

Ethical approval of the study was obtained from the IRB committee in Ministry of Health, North Kordofan State, Sudan. Informed consent was given by all participants. The study was carried out following the relevant ethical guidelines and regulations.

## TRANSPARENCY STATEMENT

The lead author Ghassan E. Mustafa Ahmed affirms that this manuscript is an honest, accurate, and transparent account of the study being reported; that no important aspects of the study have been omitted; and that any discrepancies from the study as planned (and, if relevant, registered) have been explained.

## Data Availability

The datasets used and/or analyzed during the current study are available from the corresponding author on reasonable request. The data are not publicly available due to issues of privacy.
